# Cardiac Sympathetic Activity differentiates Idiopathic and Symptomatic Rapid Eye Movement Sleep Behaviour Disorder

**DOI:** 10.1038/s41598-018-25547-w

**Published:** 2018-05-08

**Authors:** Lucie Barateau, Isabelle Jaussent, Régis Lopez, Elisa Evangelista, Sofiene Chenini, Meriem Benkiran, Denis Mariano-Goulart, Yves Dauvilliers

**Affiliations:** 1 National Reference Network for Narcolepsy, Sleep-Wake Disorders Center, Department of Neurology, Gui-de-Chauliac Hospital, Montpellier, Cedex 5 France; 2 INSERM, University of Montpellier, Neuropsychiatry: Epidemiological and Clinical Research, Montpellier, France; 3 Department of Nuclear Medicine, Montpellier University Hospital, Montpellier, Cedex 5 France; 4 PhyMedExp, University of Montpellier, INSERM U1046, CNRS UMR 9214, Montpellier, Cedex 5 France

## Abstract

The pathophysiology of rapid eye movement sleep behavior disorder (RBD) associated with narcolepsy type 1 (NT1) is still poorly understood, potentially distinct from idiopathic RBD (iRBD), but may share affected common pathways. We investigated whether MIBG cardiac uptake differs between iRBD and NT1 comorbid with RBD. Thirty-four patients with NT1-RBD and 15 patients with iRBD underwent MIBG cardiac scintigraphy. MIBG uptake was measured by calculating the early and delayed heart to mediastinum (H/M) ratios. A delayed H/M ratio lower than 1.46 was considered abnormal based on a population of 78 subjects without neurological or cardiac diseases. Patients with iRBD were older, had an older RBD onset age and higher REM sleep phasic and tonic muscular activities than NT1-RBD. Lower delayed and early H/M ratios were associated with iRBD, but not with NT1-RBD, in crude and adjusted associations. The delayed H/M ratio differed between iRBD and controls, after adjustment, but not between patients with NT1-RBD and controls. In conclusion, the MIBG cardiac uptake difference between NT1-RBD and iRBD supports the hypothesis of different processes involved in RBD pathogenesis, providing for the first time a cardiac biomarker to differentiate those disorders.

## Introduction

Rapid eye movement (REM) sleep behavior disorder (RBD) is characterized by repeated and often violent episodes of dream-enacting behaviors that may cause injury or sleep disruption^1,2^. Preclinical and clinical evidences indicate that RBD results from the breakdown of the brainstem signaling network underlying REM sleep atonia, with an excess of muscle activity during REM sleep^3,4^. RBD can be idiopathic (iRBD) or secondary. Secondary RBD can be associated with neurodegenerative disorders, especially synucleinopathies, narcolepsy, but also brainstem lesions, Guillain-Barré syndrome, intake of some drugs, and alcohol withdrawal^2^. Conversely, iRBD is not associated with other neurological diseases, but often precedes the development of synucleinopathies.

The presence of RBD is frequently associated with autonomic dysfunction in both idiopathic and secondary form associated with Parkinson’s disease (PD). Cardiac ^123^I-labeled meta-iodobenzylguanidine (MIBG, a physiological norepinephrine analogue) scintigraphy is used to assess the function of postganglionic presynaptic cardiac sympathetic nerve endings. MIBG cardiac uptake is markedly decreased in patients with iRBD in the same range as in PD and dementia with Lewy bodies, and could be used as an early biomarker of iRBD^5,6^. However, it is unclear whether and to which extent MIBG cardiac accumulation is impaired in the presence of secondary RBD outside the context of neurodegenerative diseases.

Narcolepsy type 1 (NT1) is a rare disease caused by the selective and irreversible loss of hypocretin neurons^7^. It is characterized by excessive daytime sleepiness (EDS), cataplexy, clinical manifestations related to REM sleep dysregulation (RBD, sleep paralysis and hypnagogic hallucinations) and frequent autonomic dysfunction^7,8^. RBD is observed in up to 60% of patients with NT1, and may be the first symptom even in children^9,10^. Differently from iRBD, RBD comorbid with NT1 does not show sex predominance (iRBD affects mostly men), movements are elementary rather than complex, behaviors are less violent, and RBD starts earlier^11^.

The pathophysiology of RBD associated with NT1 is still poorly understood, highly probably distinct from that of iRBD, but may share affected common pathways in both conditions. Hypocretin deficiency could cause autonomic dysfunction, a functional defect in the motor control involved in the development of cataplexy during wakefulness and of RBD during sleep^3,11^. However, recent studies showed that the brain functional connectivity during RBD episodes seems similar between patients with iRBD and RBD associated with NT1 or PD^12^. Moreover, few studies reported the development of PD in patients with NT1^13–16^, but the role of RBD in this association is unknown, and no study has assessed the frequency of synucleinopathies among patients with NT1. So far, it is not known whether RBD is a predictor for neurodegeneration in NT1.

Despite clear differences in phenotype between patients with iRBD and NT1 comorbid with RBD, we aimed in this study to investigate whether the ^123^I-MIBG cardiac uptake profiles can differentiate patients with RBD whether idiopathic or symptomatic in the context of NT1.

## Results

Compared with the NT1-RBD group, patients with iRBD were older, and also had older age of RBD onset, longer sleep-onset at night and REM sleep latency. Patients with iRBD displayed higher REM sleep phasic and tonic EMG activities. Specifically, 80% of them had tonic chin EMG density ≥30% or phasic chin EMG density ≥15%, compared with 35.3% of patients with NT1-RBD **(**Table 1). Clinical RBD episodes were documented by vPSG recording in all patients with iRBD, but only in 57.1% of patients with NT1-RBD. Among iRBD patients, eight had an obstructive sleep apnea syndrome (OSAS): three severe OSAS (AHI ≥30/h) and five moderate OSAS (AHI ≥15 and <30/h). All severe and one moderate OSAS patient were treated by continuous positive airway pressure (C-PAP) at time of scintigraphy. Among NT1 patients, eleven had OSAS (seven severe and four moderate OSAS), but only four patients were treated by C-PAP at time of scintigraphy, as this investigation was performed the day after PSG in most of NT1 patients.

**Table 1:** Clinical and polysomnographic characteristics of patients with narcolepsy type 1 (NT1) and rapid eye movement sleep behavior disorder (RBD), and of patients with idiopathic RBD (iRBD).

Variable	NT1 with RBD N = 34		iRBD N = 15		
	n	%	n	%	p-value
**Clinical characteristics**					
Sex (Male)	25	73.53	12	80.00	0.73
Age at evaluation, years^a^	51.00 (15.00; 86.00)		72.00 (48.00; 82.00)		0.0002
BMI, kg/m^2a^	26.52 (17.00; 47.80)		25.40 (22.28; 33.80)		0.19
BMI, kg/m^2^ (≥30)	8	23.53	2	13.33	0.70
Cardiovascular and metabolic comorbidities^b^ (yes)	20	58.82	10	66.67	0.60
Age of RBD onset, years^a^	36.00 (15.00; 65.00)		62.00 (47.00; 76.00)		<0.0001
RBD duration, years^a^	9.50 (0.50; 40.00)		6.00 (1.00; 15.00)		0.65
Daily RBD episodes (yes)	7	20.6	5	33.33	0.34
NT1 duration, years^a^	17.00 (0.50; 69.00)		—		
**Nocturnal sleep characteristics**					
Total Sleep Time, minutes^a^	369.50 (164.00; 522.00)		381.00 (229.00; 445.00)		0.81
Night Sleep Latency, minutes^a^	5.00 (0.00; 38.00)		31.00 (4.00; 106.00)		<0.0001
Night REM Sleep Latency, minutes^a^	55.00 (0.00; 328.00)		119.00 (35.00; 234.00)		0.03
Sleep efficiency, %^a^	77.81 (35.61; 95.55)		76.87 (50.90; 93.00)		0.65
% Stage 1^a^	11.06 (2.52; 31.70)		7.99 (1.56; 16.25)		0.04
% Stage 2^a^	47.68 (32.80; 78.05)		57.54 (32.89; 67.09)		0.17
% Stage 3^a^	14.93 (0.00; 38.19)		12.04 (0.85; 38.99)		0.95
% REM sleep^a^	22.78 (1.81; 37.62)		20.05 (3.11; 30.89)		0.48
AHI /hour^a^	6.65 (0.00; 59.39)		10.21 (0.00; 37.77)		0.78
PLMS index, /hour^a^	8.52 (0.20; 161.89)		5.00 (0.00; 154.28)		0.47
Micro-arousal index, /hour^a^	21.95 (3.30; 68.57)		21.71 (2.23; 37.69)		0.92
Mean Oxygen Saturation %^a^	95.00 (87.00; 97.00)		94.00 (91.00; 96.00)		0.51
**REM sleep without atonia**					
Phasic EMG activity %^a^	9.15 (1.50; 31.67)		20.60 (2.86; 67.80)		0.006
Phasic EMG activity % (≥15%)	9	26.47	12	80	0.002
Tonic EMG activity %^a^	19.15 (0.70; 96.43)		38.57 (1.25; 95.00)		0.02
Tonic EMG activity (≥30%)	10	29.41	9	60	0.18
Phasic (≥15%) or Tonic (≥30%) EMG activities	12	35.29	12	80	0.01

^a^Continuous variables are expressed as median (minimal value; maximal value).

*REM = rapid eye movement*, *BMI = body mass index*, *AHI = apnea hypopnea index*, *PLMS = periodic leg movement during sleep*, *EMG = electromyography*, *MSLT = multiple sleep latency test*, *SOREMPs = sleep onset REM periods*.

^b^Cardiovascular and metabolic comorbidities: Presence of diabetes type 2 or use of antidiabetic drugs, cardiac disorders (personal history of coronary artery disease, chronic heart failure, arrhythmia), hypertension or treatment with antihypertensive drugs, dyslipidemia or treatment with lipid-lowering drugs, or stroke.

Overall, 58.8% of the NT1 patients with RBD and 66.7% of the iRBD patients had a cardiovascular or metabolic comorbidity, without significant difference between the two groups (Table 1). Among iRBD patients, eight were treated with anti-hypertensive drugs (including two beta-blockers), and one with an antiarrhythmic drug at time of scintigraphy. Among NT1 patients, nine were treated with anti-hypertensive drugs (including two beta-blockers). Other patients were drug-free for cardiovascular or antihypertensive drugs at time of scintigraphy.

**Figure 1:**
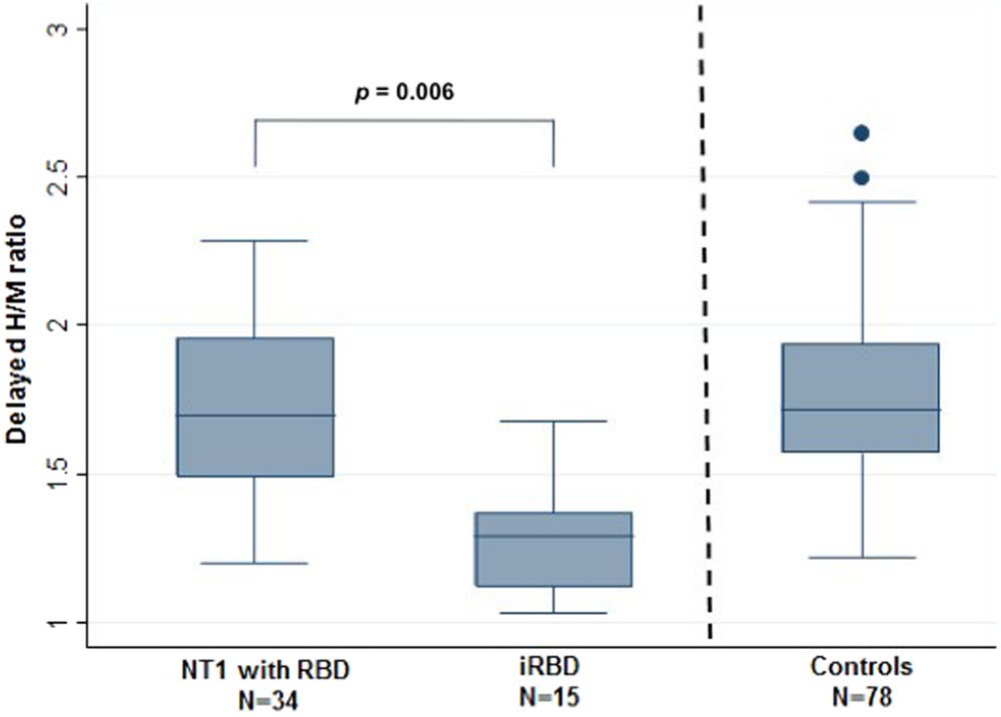
Delayed heart-to-mediastinum (H/M) ratio in patients with narcolepsy type 1 (NT1) with rapid eye movement sleep behavior disorder (RBD), in patients with idiopathic RBD (iRBD) and in controls. Results are shown as box-whisker plots with the median and 25th quartile of the delayed H/M ratio.

In crude and age-adjusted associations, lower cardiac MIBG uptake (i.e., lower delayed and early H/M ratios) was associated with iRBD, whereas the MIBG washout values were comparable in both groups **(**Table 2, Fig. 1**)**. When the H/M ratio values were divided in tertiles, the lowest tertile (H/M ratio <1.40) was associated with iRBD, but not with NT1-RBD, in crude and adjusted associations **(**Table 2**)**.

**Table 2:** Comparison of cardiac scintigraphy results in patients with narcolepsy type 1 (NT1) and rapid eye movement sleep behavior disorder (RBD) and in patients with idiopathic RBD (iRBD).

Variable	NT1 with RBD n = 34		iRBD n = 15		Model 0			Model 1	
	n	%	n	%	OR [95% CI]		p	OR [95% CI]	p
Delayed H/M ratio^a,b^	1.70 (1.20; 2.29)		1.29 (1.03; 1.68)		2.32 [1.42–3.80]		0.0008	2.07 [1.23–3.49]	0.006
**Delayed H/M ratio**									
<1.40^c^	4	11.76	12	80.00	30.00 [5.82–154.63]		<0.0001	14.02 [2.31–85.03]	0.004
≥1.40	30	88.24	3	20.00	1			1	
Early H/M ratio^a,b^	1.64 (1.40; 2.29)		1.35 (1.08; 1.73)		3.05 [1.49–6.24]		0.002	2.44 [1.19–5.00]	0.02
MIBG washout %^a,d^	32.00 (24.00; 50.00)		38.00 (24.00; 56.00)		2.02 [0.85–4.79]		0.11	1.21 [0.46–3.19]	0.70

^a^Continuous variables are expressed as median (minimal value; maximal value).

^b^OR for 0.10-unit decrease.

^c^Lowest tertile of the sample *vs* the other two.

^d^OR for 10-unit decrease.

Model 0: crude association.

Model 1: adjustment for age.

In patients with iRBD, the delayed H/M ratio was not correlated with age, body mass index (BMI), RBD frequency, PLMS and AHI indexes, and REM sleep tonic and phasic EMG activities. The sensitivity analysis found no difference in the delayed H/M ratio in drug-free and treated (clonazepam) patients with iRBD (n = 9 vs n = 6).

In patients with NT1-RBD, the delayed H/M ratio was negatively correlated with age (r = −0.48; *p* = 0.004), BMI (r = −0.53; *p* = 0.001), and percentage of RSWA activity (r = −0.4; *p* = 0.02), but not with RBD frequency, phasic REM sleep EMG activities, PLMS and AHI indexes, and CSF hypocretin-1 levels. All associations, except for age, remained unchanged when the NT1-RBD subgroup of narcolepsy-drug-free patients at scintigraphy only (n = 21, 71% men, median age: 34 years, delayed H/M = 1.83 [1.46–2.29]) was analyzed. In addition, the delayed H/M ratio was higher in the drug-free (n = 21) than treated (psychostimulants and anticataplectic agents) NT1-RBD group (n = 13) (*p* = 0.002). However, this difference was not significant after adjustment for age.

In the control group (n = 78), the first decile of delayed H/M ratio was 1.46. Using this threshold to define abnormal values, thirteen (86.7%) patients with iRBD and four patients with NT1-RBD (11.8%) had pathological scintigraphy results. These four NT1-RBD patients with low H/M ratio (3 men and one woman, range 56–84 years old, BMI 26–48, delayed H/M 1.20–1.33, EMG phasic activity 5.5–27.5%, EMG tonic activity 1.9–94%) had a treatment for narcolepsy (psychostimulants and anticataplectic). Three of them had a cardiovascular comorbidity, three received antihypertensive drugs (but not beta-blockers), three had a non-dipping blood pressure profile, and two severe OSAS (including one using a CPAP at time of MIBG). None of these four patients had prodromal symptoms of Lewy body pathology: no subtle parkinsonian signs, no olfactory loss, and no constipation.

The delayed H/M ratio was significantly different between patients with iRBD and controls (H/M = 1.29 [1.03–1.68] vs 1.72 [1.22–2.65], *p* < 0.0001). This difference persisted after adjustment for age and sex (*p* = 0.0007). Conversely, the delayed H/M ratio was not different between patients with NT1-RBD and controls in crude and adjusted analyses (delayed H/M = 1.70 [1.20–2.29] vs 1.72 [1.22–2.65], *p* = 0.82).

## Discussion

This study reports significant differences in cardiac ^123^I-MIBG uptake between patients with iRBD and patients with NT1 and comorbid RBD. The threshold to define pathological cardiac MIBG values is challenging. Several cutoffs, from 1.4 to 2.8, were reported in the literature, with differences related to the involved populations, sample size, procedure and scanning methods (i.e., the used collimator), and statistical analyses^17^. Here, we defined the abnormal levels based on a control population of 78 subjects without neurological or cardiac disorders classified in quantiles, with low values below the lowest decile of 1.46 (local clinical normal values). We found that 87% of patients with iRBD, but only 12% of patients with NT1-RBD had abnormal H/M ratios. This supports the hypothesis of different pathophysiological processes between iRBD and NT1-associated RBD, an hypothesis that was predictable, but never proven, and consequently implies differences in the risk of conversion to neurodegenerative disorders.

Abnormal ^123^I-MIBG cardiac scintigraphy results are currently considered as a strong biomarker of synucleinopathies^18^. Moreover, few studies showed cardiac sympathetic dysfunction even at early stages of iRBD^5,6,19,20^. Specifically, compared with controls and also patients with multiple system atrophy or progressive supranuclear palsy, early and delayed H/M ratios are reduced in patients with iRBD to a similar extent as in patients with PD or dementia with Lewy bodies.

A study reported a correlation between iRBD duration and H/M ratio^6^. Similarly, in one of our patients with iRBD (onset age: 47 years), the delayed H/M ratio decreased over time (H/M = 1.35 at 62 years of age, at the time of study inclusion, 1.23 at 65 years, and 1.21 at 67 years). Other studies showed a more marked reduction of MIBG cardiac uptake in iRBD compared with early PD stages^19^, and also in PD with RBD compared with PD without RBD^20,21^. These results suggest a more complex contribution of cardiac sympathetic dysfunction in the context of RBD. Postganglionic cardiac sympathetic denervation (i.e., loss of tyrosine hydroxylase-positive nerve fibers) is frequent in PD, and Lewy body (alpha-synuclein-positive) accumulation in the peripheral autonomic system precedes the neuronal loss in sympathetic ganglia^22^. In PD, decreased MIBG uptake reflects the cardiac sympathetic denervation and differences in MIBG accumulation depend on the injury level. Therefore, RBD could modify the extent of cardiac autonomic nerve denervation independently of motor symptoms. Accordingly, patients with iRBD often have orthostatic hypotension and cardiac dysfunction during wakefulness and sleep, with similar patterns observed in patients with PD and RBD compared with patients with PD without RBD^20,21,23^. Moreover, cardiac autonomic dysfunction assessed by RR variability or MIBG may not predict the risk of neurodegenerative disease in patients with iRBD^20,21,23^. Here, we found no correlation between the delayed H/M ratio and AHI indexes in both iRBD and NT1-RBD populations, as previously reported^24^. The mechanism by which RBD is strongly associated with sympathetic alteration remains mostly unknown.

RBD was reported in 45 to 61% of patients with NT1^11^, with recent findings showing its presence also in children even before sleepiness and cataplexy onset^9,10^. The best RBD biomarkers are reduction of REM sleep atonia and excessive phasic REM sleep muscle activity. These parameters have been largely studied in iRBD and thresholds have been defined to differentiate patients and controls^25–27^. However, no cut-off is currently available in the context of RBD associated with NT1^28–30^. Here, we found increased REM sleep phasic and tonic EMG activities in patients with NT1-RBD, but to a lesser extent than in the iRBD group.

Pathophysiology of RBD whether idiopathic or symptomatic in the context of NT1 remains unclear, is often associated with autonomic dysfunction, and thus could have shared an alteration of common pathways under both conditions. Accordingly, recent ictal single-photon emission tomography studies explored the RBD pathways *in vivo* and showed similar activation in the bilateral premotor areas, interhemispheric cleft, periaqueductal area, dorsal and ventral pons and the anterior lobe of the cerebellum in patients with iRBD and in those with RBD associated with PD or NT1^12,31,32^. Conversely, the significantly lower percentage of patients with NT1-RBD with abnormal MIBG H/M ratio (12% vs 87% in the iRBD group) in our study suggests a different pathophysiological origin. In NT1, cardiac tissue involvement has never been formally proved, although recent studies reported some alterations in sympathetic activities. Patients with NT1 have decreased resting muscle sympathetic nerve activity, heart rate, and blood pressure during wakefulness^33^, and frequent non-dipper blood pressure profile associated with REM sleep dysregulation^34^. Several preclinical and clinical evidences also support a direct effect of hypocretin on autonomic regulation^35^. Hypocretin deficiency in NT1 could lead to dissociated REM sleep features, including REM sleep without atonia, increased density of phasic chin EMG during REM sleep, frequent shift from REM to non-REM sleep and wake, and could also trigger RBD^36^. However, all patients with NT1 were hypocretin deficient but only half of them will develop RBD^12^. In addition, the delayed MIBG H/M ratio did not correlate with CSF hypocretin-1 levels, but with the percentage of RSWA activity; a result in line with a recent study showing that CSF hypocretin-1 levels are normal in a small group of iRBD patients^37^.

The association between NT1 and synucleinopathies remains controversial, with few available case-report studies^13–16^, and RBD involvement in this association is unknown. As NT1 is an orphan disease, often starting in adolescent or young adults^38^, it is difficult to conduct a prospective study in a large NT1 cohort to assess the incidence of synucleinopathies in the right age group. Based on our results, we plan to clinically follow very closely the selected group of 4 NT1-RBD patients with abnormal MIBG scintigraphy, to assess on a potential development of synucleinopathies.

The present study has some limitations. The low sample size of patients with iRBD and NT1, the frequent cardiovascular and metabolic comorbidities and the frequent drugs intake for narcolepsy and cardiovascular disorders may theoretically influence MIBG results. However, the hypothetical effect of those medications would be to decrease H/M ratio, and thus increase the association between NT1-RBD and reduced MIBG accumulation, but we did not find such significant differences. Finally, as we included in this study only patients with RBD, a further MIBG scintigraphy study in a larger population of patients with NT1 with and without associated RBD compared to matched control subjects is required to evaluate the autonomic cardiac activity and identify patients with abnormal function.

To conclude, the MIBG cardiac uptake difference between patients with NT1-RBD and with iRBD supports the hypothesis of different processes involved in RBD pathogenesis, providing for the first time a cardiac biomarker to differentiate those disorders.

## Methods

### Participants

We included 34 patients with NT1 and comorbid RBD (NT1-RBD) (25 males, median age 51 years, range 15–86) at the Reference National Center for Narcolepsy of Montpellier, France. NT1 was diagnosed according to the ICSD-3 criteria^1^: presence of EDS, mean sleep latency <8 minutes on the Multiple Sleep Latency Test (MSLT), at least two sleep-onset REM periods on MSLT or night polysomnography (PSG), cataplexy or low cerebrospinal fluid (CSF) hypocretin-1 levels (<110 pg/mL). CSF hypocretin-1 level was measured in 25 patients (74%) and was low in all of them. All patients were positive for HLA DQB1*06:02. RBD was defined according to the ICSD-3 criteria^1^: history of injurious or disruptive sleep behaviors with dream enactment, documentation of their occurrence during REM sleep by video-PSG (vPSG), and demonstration of REM sleep without atonia (RSWA) by PSG.

Fifteen patients with a diagnosis of iRBD according to the ICSD-3 criteria^1^ (12 males, median age 72 years, range 48–82) were also recruited. They all had a clinical history of dream-enactment behaviors and vPSG documentation of RBD.

Both groups of patients were carefully examined by a neurologist to exclude any association with neurological disorders (including extra-pyramidal symptoms and history of stroke).

The institutional review boards of the University of Montpellier-France approved this study. The methods were carried out in accordance with the approved guidelines. Each participant signed legal consent forms. Informed consent was obtained from all subjects.

A control clinical population recruited through the Medicine Nuclear Department of the University Hospital of Montpellier was included to determine the normal values for MIBG parameters, as threshold to define pathological cardiac MIBG values often differed in the literature, mainly due to the involved populations and procedure and scanning methods^17^. This group of 78 subjects (36 males, median age 53.5 years, range 10–84) had no neurological or cardiac disorders, no PSG assessment, and underwent ^123^I-MIBG cardiac scintigraphy for another reason, mostly to exclude a pheochromocytoma.

### Polysomnography

All patients underwent one-night vPSG recording in the sleep laboratory. Sleep stages were manually scored as well as micro-arousals, periodic limb movements during sleep (PLMS) and apnea-hypopnea index (AHI), according to standard criteria^39^. REM sleep was scored without the chin electromyographic (EMG) criterion allowing for the maintenance of muscle tone during REM sleep. A 30-second-epoch of REM sleep was defined as tonic if tonic chin EMG activity was present for ≥50% of the epoch. Phasic EMG activity was scored from the chin EMG recording and represented the percentage of 2-second mini-epochs of REM sleep containing phasic EMG events, defined as any burst of muscle activity lasting from 0.3 to 5 seconds with amplitude that exceeded four times the baseline EMG signal. RSWA was defined as the presence of a tonic REM activity that exceeded 30% of the total REM sleep duration, and phasic EMG activity was considered abnormal when higher than 15%^27^.

### Cardiac ^123^I-MIBG scintigraphy

MIBG myocardial scintigraphy is a nuclear imaging technique that can evaluate the cardiac sympathetic function at a clinical level. It is a very sensitive measure of the post-ganglionic sympathetic activity of the myocardium. It provides useful information for evaluation of disease severity, prognosis, and therapeutic effects; in particular in patients with heart failure, ischemic heart diseases, or arrhythmic disorders. The heart to mediastinum ratio (H/M ratio) and the washout rate are the most common indices.

All patients underwent at rest myocardial sympathetic innervation scintigraphy using a General Electric INFINIA Hawkeye 4 gamma camera (GE Healthcare, Tirat Carmel, Israel) with low energy, high resolution collimators^40^. Anterior planar 64 × 64 pixel images were acquired at 10 minutes (early phase) and 3 hours (delayed phase) after intravenous administration of ^123^I-MIBG (185 MBq). Thyroid was blocked by oral administration of 130 mg of potassium iodine 1 hour before ^123^I-MIBG injection^41^. The early and delayed (i.e., the most reliable MIBG biomarker) heart to mediastinum (H/M) uptake ratios were calculated by dividing the mean count in a manually-drawn, left ventricular region of interest (ROI) by the mean count in a 7 × 7-pixel ROI square placed in the upper mediastinum to avoid lung activity. The MIBG washout rate (i.e., the rate of MIBG washed out between the early and the delayed images) was calculated by comparing the cardiac count in the ventricular ROI of the early and delayed images^41^. The physicians who analyzed the scintigraphic data (DMG, MB) and calculated the delayed and early H/M ratios and MIBG washout rate were blinded to the diagnosis. Scintigraphy was performed after vPSG recording, with a median delay of 1 day (range 0–1830) for patients with NT1-RBD and 127 days (range 0–990) for patients with iRBD.

### Cardiovascular and metabolic comorbidities, and treatment intake

Cardiovascular and metabolic comorbidities were defined as the presence of diabetes type 2 or use of antidiabetic drugs, cardiac disorders (personal history of coronary artery disease, chronic heart failure, arrhythmia), hypertension or treatment with antihypertensive drugs, dyslipidemia or treatment with lipid-lowering drugs, or stroke.

At the time of the scintigraphy, thirteen (38.2%) patients with NT1-RBD were taking drugs (psychostimulants: n = 12; anticataplectic agents: n = 9 (2 a selective serotonin-reuptake inhibitor (SSRI, fluoxetine), 5 a serotonin and norepinephrine reuptake inhibitor (SNRI, venlafaxine), and 2 a tricyclic antidepressant (clomipramine); sodium oxybate: n = 2); and six (40%) patients with iRBD were taking benzodiazepines (clonazepam) at low dose, and none antidepressants.

### Statistical analysis

Categorical variables were presented as percentages, and quantitative variables as medians with ranges. Quantitative variables were mostly skewed according to the Shapiro-Wilk test. Demographic and clinical characteristics between patients with NT1-RBD and patients with iRBD were compared using the Chi-square or Fisher’s exact test (for categorical variables) and the Mann-Whitney test (for continuous variables) (Table 1). To study the relationship between cardiac scintigraphy data and the two groups of patients, logistic regression models were used to estimate the odds-ratios (OR) and their 95% confidence interval (95% CI) (Table 2). Model 1 showed the non-adjusted association between cardiac scintigraphy variables and the two groups and model 2 the age-adjusted association. ORs evaluated the strength of the associations between cardiac scintigraphy variables and the two groups. Spearman’s rank order correlations were used to determine associations between continuous variables (*i*.*e* delayed ratio with continuous demographic and clinical characteristics such as age and BMI). The significance level was set at p < 0.05. Analyses were performed with the SAS software (version 9.4; SAS, Cary, NC).
